# Transcriptome analysis of cattle muscle identifies potential markers for skeletal muscle growth rate and major cell types

**DOI:** 10.1186/s12864-015-1403-x

**Published:** 2015-03-13

**Authors:** Bing Guo, Paul L Greenwood, Linda M Cafe, Guanghong Zhou, Wangang Zhang, Brian P Dalrymple

**Affiliations:** Key Laboratory of Meat Processing and Quality Control, Synergetic Innovation Centre of Food Safety and Nutrition, College of Food Science and Technology, Nanjing Agriculture University, Nanjing, 210095 P. R. China; CSIRO Agriculture Flagship, St. Lucia, QLD 4067 Australia; CSIRO Agriculture Flagship, Armidale, NSW 2350 Australia; NSW Department of Primary Industries, University of New England, Armidale, NSW 2351 Australia

**Keywords:** Adipogenesis, ADG, Angiogenesis, Cattle muscle, Development, Extracellular matrix

## Abstract

**Background:**

This study aimed to identify markers for muscle growth rate and the different cellular contributors to cattle muscle and to link the muscle growth rate markers to specific cell types.

**Results:**

The expression of two groups of genes in the longissimus muscle (LM) of 48 Brahman steers of similar age, significantly enriched for “cell cycle” and “ECM (extracellular matrix) organization” Gene Ontology (GO) terms was correlated with average daily gain/kg liveweight (ADG/kg) of the animals. However, expression of the same genes was only partly related to growth rate across a time course of postnatal LM development in two cattle genotypes, Piedmontese x Hereford (high muscling) and Wagyu x Hereford (high marbling). The deposition of intramuscular fat (IMF) altered the relationship between the expression of these genes and growth rate. K-means clustering across the development time course with a large set of genes (5,596) with similar expression profiles to the ECM genes was undertaken. The locations in the clusters of published markers of different cell types in muscle were identified and used to link clusters of genes to the cell type most likely to be expressing them. Overall correspondence between published cell type expression of markers and predicted major cell types of expression in cattle LM was high. However, some exceptions were identified: expression of *SOX8* previously attributed to muscle satellite cells was correlated with angiogenesis. Analysis of the clusters and cell types suggested that the “cell cycle” and “ECM” signals were from the fibro/adipogenic lineage. Significant contributions to these signals from the muscle satellite cells, angiogenic cells and adipocytes themselves were not as strongly supported. Based on the clusters and cell type markers, sets of five genes predicted to be representative of fibro/adipogenic precursors (FAPs) and endothelial cells, and/or ECM remodelling and angiogenesis were identified.

**Conclusions:**

Gene sets and gene markers for the analysis of many of the major processes/cell populations contributing to muscle composition and growth have been proposed, enabling a consistent interpretation of gene expression datasets from cattle LM. The same gene sets are likely to be applicable in other cattle muscles and in other species.

**Electronic supplementary material:**

The online version of this article (doi:10.1186/s12864-015-1403-x) contains supplementary material, which is available to authorized users.

## Background

The development and growth of skeletal muscle is a complex process, involving not just the muscle contractile cells, but also the expansion of the extracellular matrix to provide support and the blood vessels to provide the oxygen and energy required [1,2]. The contractile component of skeletal muscle increases in mass through two processes. In prenatal development, and very early post natal development in some species, muscle fibres grow by increase in fibre numbers (hyperplasia). Once that phase of growth has been completed further growth of muscle occurs without increase in fibre numbers, rather the volume of fibres increases (hypertrophy), with increase in fibre area and length [3]. To date, most studies, such as muscle fibre ontogenesis through development [4], mechanism of myogenesis [5-8] and endocrine and metabolic regulation [9], have focused on the main components of skeletal muscle, the muscle contractile cells. However, skeletal muscle also contains a considerable population of fibroblasts, intramuscular adipocytes, and other cell types including nerve cells and blood vessels [3]. All of these components contribute to skeletal muscle growth, most likely in a complex molecular crosstalk between the cell types [2].

The rate of increase of muscle mass is a key parameter in livestock production. Faster growing animals are generally more efficient as proportionally less energy is required for maintenance. However, the rate of increase in muscle mass, or average daily gain (ADG) of muscle cannot be directly measured on a live animal. Change in eye (longissimus) muscle (LM) area (EMA) is an estimator of the growth of muscle, but EMA is generally measured post slaughter and is expensive to measure on live animals, requiring ultrasound scanning [10]. It is also not an accurate estimator of muscle volume. However, during allometric growth of an animal the growth of skeletal muscle is assumed to be proportional to the growth of the whole animal [11]. Thus ADG of muscle is generally proportional to ADG of the whole animal. The measurement of the weight of an animal is relatively simple, although multiple measurements are required over several days to estimate animal ADG accurately. This is particularly true in large animals, such as cattle, where the variable mass of gut contents and water leads to large variation in measured weight unrelated to actual animal weight. Thus the growth rate of an animal, and more importantly the growth rate of its muscle cannot be accurately estimated from just one measurement. Analysis of gene expression may enable us to identify a set of genes suitable for the estimation of ADG muscle/kg liveweight similar to the use of gene expression to estimate the rate of deposition of intramuscular fat (IMF) [12]. In addition, increasing our understanding the cellular and molecular mechanisms of cattle skeletal muscle growth will be important for the development of approaches for the manipulation of the commercially important phenotype, average daily gain (ADG) of muscle [5,13]. By defining robust sets of genes representing the outputs of biological processes we can apply tools such as regulatory impact factor analysis (RIF) [14] and module to regulator analysis [15] to identify the drivers of the outputs.

In the current work, we have used the correlation between ADG/kg liveweight and gene expression profiles from the LM of three different beef cattle breeds/genotypes to identify genes whose expression is correlated with the growth rate of cattle muscle. In order to maximise the information content of the analysis the animals chosen have variation in IMF% (ranging from 2% to 9%), ages, growth rates and diets and were with/without Hormone Growth Promotant (HGP)-treatment. Using these genes we have applied a number of bioinformatics approaches to identify the most likely population(s) of cells in muscle expressing them. As part of this analysis we have also investigated the relationship between published markers of cell types present in skeletal muscle and the clustering of gene expression patterns across development. By combining the two approaches we have identified a number of small sets of genes likely to be representative of Fibroadipogenic precursors (FAPs) and endothelial cells, and potentially currently unidentified subsets of these cells and/or ECM remodelling and angiogenesis.

## Results and discussion

### Correlation of gene expression with ADG/kg liveweight

The full set of genes included on the microarray were ranked based on the correlation between gene expression in the LM and the ADG/kg liveweight of the same individual across 48 Brahman cattle (Additional file 1: Table S1). To identify biological processes enriched towards the top of the ranked list of genes, the list was submitted to GOrilla [16]. The GO terms with significant *Q*-values (less than 10^−5^) and additional terms with less significant enrichment containing sets of genes likely to be involved in LM development based on the current knowledge of muscle development were identified (Table 1). Correlation analysis between gene expression and a phenotype generally identifies the output genes, rather than the regulators of the output [17]. That is the genes identified are likely to be good indicators of the rate of animal/muscle growth, but are less likely to be the key drivers of muscle growth rate. Indeed, some of the key drivers, such as growth hormone, are synthesized elsewhere in the body and hence not analysed at all in the LM. The expression of the gene encoding the muscle mass regulator, myostatin (MSTN) [18], which would be expected to be negatively correlated with ADG/kg liveweight, had a correlation coefficient of −0.49, and expression of the gene associated with the callipyge highly muscling phenotype in sheep, *DLK1* [19], which would be expected to be positively correlated had a positive correlation, 0.43 (Additional file 1: Table S1). But neither of these values was significant. The correlation of another gene encoding a protein known to be involved in muscle growth, IGF1, which would be expected to be positively correlated, is much higher (0.72), ranked 117th of all genes (Additional file 1: Table S1). However, given that the data is noisy such results involving a single gene should be treated with caution, and we have not explored them further in this work. In addition, the muscle contractile cells are the major cellular component of the tissues, and thus the major contributor of the gene expression signal. Therefore the normalization of the gene expression data will have tended to reduce between sample differences in gene expression of genes predominantly expressed in the muscle contractile cells. That is, genes whose expression is correlated with global transcriptional changes in the contractile cells will be harder to detect in this analysis, particularly in the post natal samples. Thus the analysis is aimed at the identification of genes whose expression is altered relative to the bulk of the expression of the contractile cells during the growth of the animals.

**Table 1:** **GO enrichment analysis of genes ranked by the correlation of gene expression with ADG/kg in 48 Brahman steers**

**GO term**	**Gene number**	**FDR Q-values**
Cell cycle process	66	2.58E^−5^
Extracellular matrix organization	46	1.24E^−4^
Axon guidance	49	1.25E^−3^
Angiogenesis	42	8.1E^−3^

### Refining the selection of genes

Contrasting data from two different datasets and analysis methodologies is an effective method of increasing the ratio of signal to noise in gene expression datasets [17]. It has advantages which cannot be matched by the application of more sophisticated analyses of a single dataset. We then investigated the expression profiles of the genes in the GO terms identified above across LM development in a high muscling genotype, Piedmontese x Hereford (PxH), and a high marbling genotype, Wagyu x Hereford (WxH). The top five genes in each GO term which satisfied the following criteria: co-expressed through development in the PxH and WxH animals, and highly correlated with ADG/kg liveweight in the Brahman dataset were defined as a gene set. As a result five genes included in the “cell cycle process” GO term, and five genes included in the “ECM organization” GO term were defined as the “cell cycle 5 gene set” and the “ECM 5 gene set”, respectively (Table 2). One of the objectives of the work was to identify a number of small and robust sets of genes for use to estimate the impact of age, treatment, genetics etc. on particular biological processes and cell types in cattle skeletal muscle. Co-expression through development was used as a selection criterion as co-expressed genes are more likely to be expressed by the same cell type, or involved in the same or very closely related biological processes [12,17], than genes which are not co-expressed.

**Table 2:** **Gene sets identified by the k-means clustering analysis**

**Gene set**	**Enriched GO term** ^**1**^	**Cluster numbers**				**5 gene set**	**Possible biological process**
		**k = 13**	**k = 10**	**k = 13**	**k = 10**		
		**All** ^**2**^	**All**	**PN** ^**3**^	**PN**		
IMF^4^	N/A	N/A	N/A	N/A	N/A	*ACSM1, CIDEA, DGAT2, FABP4, THRSP*	IMF deposition
ECM^5^	ECM organization	N/A	N/A	N/A	N/A	*ADAMTS4, BGN, COL5A2, TGFB2, SERPINH1*	General ECM remodelling
Cell cycle	Cell cycle process	C9	C3	C9	C5	*CDC6, CDC20, CDCA3, KIF20A, KIF23*	General mitotic cell division
ECM1	ECM organization	C9	C3	C9	C5	*COL5A2, COL1A2, SDC3, SH3PXD2B, TNC*	General ECM remodelling
ECM2	ECM organization	C8	C10	C10	N/A	*COL3A1, GFOD2, LAMA4, MMP15, TGFB3*	ECM remodelling involved in angiogenesis
Angiogenesis	Angiogenesis	C8	C10	C10	C5	*CCBE1, ELK3, NOTCH1, SOX18, VASH1*	Angiogenesis
Locomotion	Regulation of locomotion	C10	C9	C1	C1	*ADORA3, ENG, FLT4, IFITM2, LMNA*	Cell migration involved in the newly formed blood vessel

^1^the source of the corresponding gene set.

^2^all developmental stages.

^3^postnatal developmental stages.

^4^this gene set was generated by our previous work [12].

^5^this gene set was generated from the correlation-based analysis.

### Combining multiple genes

Gene expression data is also inherently noisy due to both technical and biological variation. Combining data from multiple genes can reduce the impact of such variation. Previous analyses using these datasets have shown that five genes is a good compromise between the number of genes and stability of the profile [12]. The individual gene expression values across the whole dataset were standardized using z-scores to enable the data from different genes to be combined. The correlation of the average z-score of the gene expression values of the five genes in the cell cycle (0.77) and ECM five gene sets (0.7) with ADG/kg liveweight in the Brahman dataset was calculated. These values were compared to the correlation between ADG/kg liveweight with 10^7^ randomly selected sets of five genes in the Brahman dataset. The results showed that there were very few randomly selected five gene combinations possessing higher correlation with ADG/kg liveweight in the 48 Brahman steers than the cell cycle (*P* < 10^−5^) and the ECM 5 (*P* < 10^−5^) gene sets.

### Expression of the cell cycle 5 gene set during development

**Figure 1:**
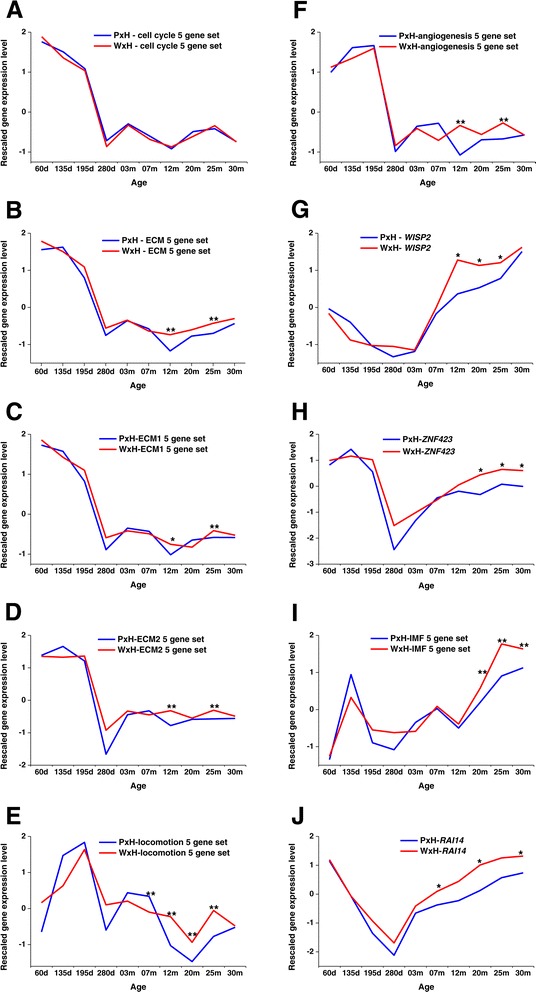
**The expression profiles of selected genes and 5 gene sets through development in PxH and WxH cattle.** Expression values of *WISP2* and *ZNF423* are z-scores. The expression levels of the 5 gene sets are the average z-scores of the 5 genes in each gene set: **A)** cell cycle 5 gene set; **B)** ECM 5 gene set; **C)** ECM1 5 gene set; **D)** ECM2 5 gene set; **E)** locomotion 5 gene set; **F)** angiogenesis 5 gene set; **G)** *WISP2*; **H)** *ZNF423*; **I)** IMF 5 gene set; **J)** *RAI14.* The symbol “*” indicates significant differences (*P* < 0.05) in expression of genes/gene sets between crosses at the same time point, and the symbol “**” indicates the significant differences at *P* < 0.01. The significance of gene sets were calculated based on z-scores of 5 members in each gene set between genotypes. The significance of individual genes was calculated based on the 95% confidence interval across all genes (more than 19,000) at the corresponding time points.

Skeletal muscle growth contributes a significant proportion of bodyweight gain after birth in vertebrates and ADG/kg generally decreases with age. However, the major components of skeletal muscle, the mature muscle fibres, do not divide and hence are unlikely to be the source of the cell cycle gene expression [3]. Thus it is likely that other populations of cells may be the source of the expression of the cell cycle genes. In the development time course ADG/kg declined from birth to 25 months with an increase from 25 to 30 months after the start of concentrate feeding [20], but cell cycle gene expression was higher at 20 and 25 months than at 12 months and decreased from 25 to 30 months (Figure 1A). Such variation suggests that the cellular origin of the expression of the cell cycle 5 gene set may change across development. In fact, muscle contains many different cell populations, several of which could be dividing and contributing to the expression of the cell cycle genes [2]. These include the muscle satellite cells, vascular/lymphatic endothelial cells, preadipocytes, fibro/adipogenic precursors (FAPs), mesenchymal stem cells and pericytes. Published analysis of the location of nuclei in three rat muscles identified: 46–64.4% in mature muscle cells, 14-25% in endothelial cells, 11-16% in fibrocytes, 2.6-4.4% in satellite cells, 4-5% in pericytes and 4-7% in other cells [21].

Since the gene expression data was from whole muscle biopsies the signal for each gene is the integration of the signal from all of the cell types expressing the gene. For genes expressed in predominantly, or only, one cell type, the gene expression signal will reflect the contribution of the cell type to the overall composition/activity of the tissue. However, for genes expressed in all, or many, cell types the signal will tend to be noisy and less well correlated with other expression signals. We have demonstrated the utility of this in our analysis of gene expression correlated with IMF deposition in the same gene expression data [12]. Below, we attempt to identify the cell populations which contribute to the variation in expression of the cell cycle genes in LM across cattle development.

### Expression of muscle satellite cell markers during development

Postnatal muscle growth is mainly due to the hypertrophy of mature contractile cells. In normal growth conditions most muscle satellite cells are quiescent [22]. However, in the postnatal animal once the limit of the ratio of contractile cell volume to myonuclei (the myonuclear domain size) is reached, additional nuclei are recruited by the fusion of muscle satellite cell derived cells with the mature contractile cells [22]. Thus satellite cell division could still contribute to the observed cell cycle gene expression. In many studies, *PAX7* and *MYF5* have been used as gene markers of muscle satellite cells [23,24]. However, their cell types of expression are not identical (Table 3), with varying proportions of *PAX7*^+^*MYF5*^−^ to *PAX7*^+^*MYF5*^+^ satellite cells observed in cattle [25] and other species [26]. The expression of *MYF5* best represents the muscle committed proliferating population and therefore is the best gene to use to describe the satellite cell division process separately from the division of other cells [27]. Despite the high muscling potential of the PxH genotype animals no significant differences in postnatal expression of *PAX7* or *MYF5* were observed between genotypes, or between time points (Additional file 2: Figure S1). Without any cell division the concentration of satellite cells, and (assuming a constant rate of expression of *PAX7* and *MYF5* in the satellite cells) the expression of *PAX7* and *MYF5*, would be expected to decrease with animal age. In order to maintain the expression of *PAX7* and *MYF5* approximately constant with age, at least a low rate of division of satellite cells would be expected. However, since the replicating satellite cells in LM may be a small proportion of the total replicating cells [21], the contribution of satellite cell division activity to the total cell cycle signal may also be small and the exact proportion of cell cycle gene expression levels derived from satellite cell division remains to be determined.

**Table 3:** **Published expression sites of selected gene markers in the major cellular components of skeletal muscle**

**Gene**							**Cell type**					
	**Pericyte type 2** **[** 33 **]**	**Self-renewing satellite cell** **[** 72 **]**	**Myogenesis committed satellite cell** **[** 72 **]**	**Myoblast** **[** 72 **]**	***Myotube*** ^***1***^	**Pericyte type 1** **[** 33 **,** 73 **]**	**Endothelial cell** **[** 73 **,** 74 **]**	**FAP** **[** 28 **]**	**Preadipocyte** **[** 75 **,** 76 **]**	***Adipocyte*** **[** 73 **,** 76 **]**	**Fibroblast** **[** 77 **]**	***Fibrocyte*** **[** 78 **]**
*NES*	**+**		**+**			**-**	**-**	**-**	**-**	**-**		
*CSPG4 (NG2)*	**+**		**-**			**+**						
*MCAM (CD146)*	**+**		**-**			**+**	**+**	**-**				
*PDGFRB*	**+**					**+**						
*PDGFRA*						**+**		**+**				
*CD34*			**+**				**+**	**+**	**+**	**-**	**-**	**+**
*SCA1*			**-**					**+**				
*PECAM1 (CD31)*							**+**		**-**	**-**		
*PAX7*	**-**	**+**	**+**	**-**	**-**	**-**						
*MYF5*		**-**	**+**	**+**	**-**	**-**						
*ITGB1 (CD29)*									**+**	**-**	**+**	
*CD24*									**+**	**-**		
*CDH5*							**+**					
*TEK*							**+**					
*SOX8*			**+**					**+?**	**+**			
*WISP2*								**+?** ^**2,3**^	**+**	**?** ^**4**^	**+** ^3^	
*ZNF423*								**+?**	**+**	**+**		
*CEBPA*									**+**	**+**		
*PPARG*									**+**	**+**		
*ACTA2*											**+**	**-**

^1^Non-dividing cells indicated in italics.

^2^[79].

^3^[48].

^4^level of expression is unclear, but see [79].

“?” represents the combination of the clustering analysis and the reference indicated above.

### Extracellular matrix producing lineages are the likely sources of cell cycle gene expression

**Figure 2:**
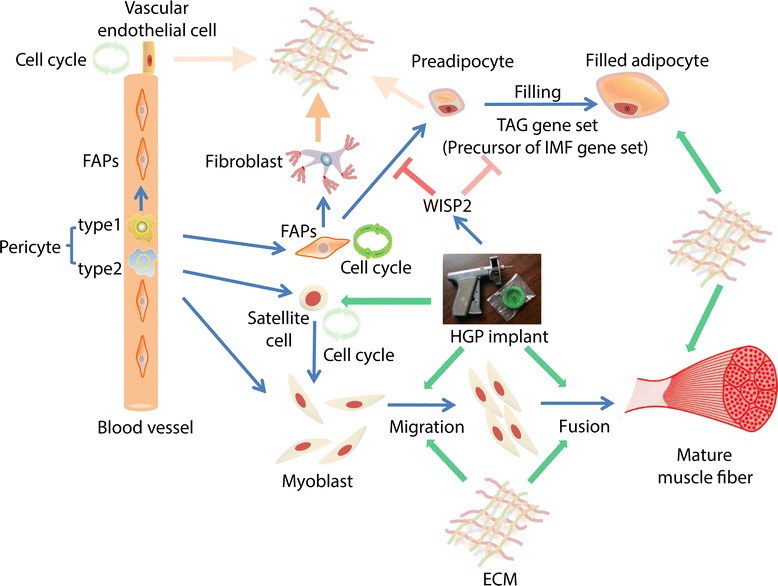
**Diagrammatic model to present the interrelationship of the main components of skeletal muscle in beef cattle.** The green arrow represents a stimulative effect. The yellow arrow represents a secretion process. The red “T” symbol represents an inhibitory effect. The shade of symbol represents the intensity of the corresponding biological function or process. The location of FAPs and pericytes, the nature of two types of pericytes and their fates, resources of ECM, genes related to the filling process of preadipocyte, as well as the effect of HGP treatment on the myogenic lineages are based on the published literature [12,17,30,31,33,65-71].

The cell cycle and ECM 5 gene sets were generally co-expressed through development in both the PxH and the WxH cattle, with similar profiles in the two groups (Figure 1A, 1B). Two significant divergences in expression of the ECM 5 gene set, higher in WxH than PxH animals, were observed between the genotypes at 12 months (*P* < 0.01) and 25 months (*P* < 0.01) of age (Figure 1B). In order to investigate the possible cell population(s) leading to these differences, based on the features of the expression profile of the ECM 5 gene set, and using loose selection criteria (expression decreasing after birth, expression higher in WxH than in PxH at 12 and 25 months), 5,596 genes were identified to be at least weakly co-expressed with the ECM 5 gene set throughout development. This set of genes was significantly enriched for ECM related GO terms (*Q* < 10^−12^). In muscle ECM is synthesized by the endothelial cells and the fibro/adipogenic progenitors (FAPs), which give rise to fibrocytes, preadipocytes and adipocytes [28,29], all of which also contribute to ECM synthesis [30,31] (Figure 2).

**Figure 3:**
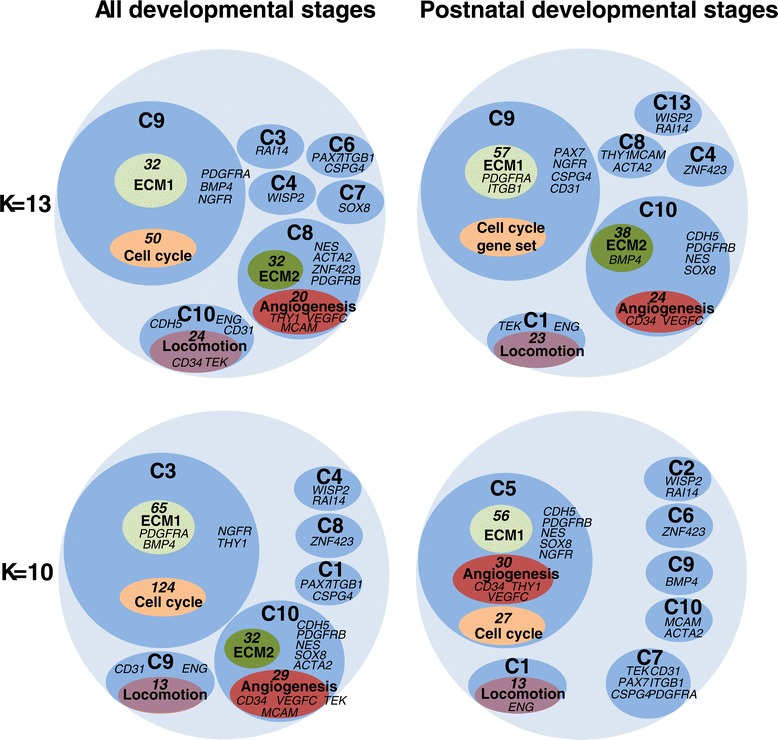
**Cluster analysis of the set of 5,596 genes co-expressed with the ECM 5 gene set.** Each light blue circle represents a separate k-means cluster analysis. Each dark blue bubble represents a cluster. The dark green, light green, dark red, light red and yellow bubbles represent the GO terms enriched in the corresponding clusters. The numbers at the top of each bubble are the number of genes within each cluster/GO term.

In order to identify the predominant cell populations and biological processes involved in ECM development, we clustered the genes based on their expression profiles through development, utilising both differences between time points within genotypes and differences between genotypes at the same time point (Figure 3). Given that there are significant differences in the expression levels of most genes between pre- and postnatal developmental stages, the k-means cluster analysis was carried out for the whole time course and for just the postnatal period. Using the whole time course maximises the discriminating power of the clustering as it contains large relative changes in expression in many genes between the pre- and postnatal time points. However, using just the post natal time points prevents major changes in gene expression relationships between the pre- and postnatal stages from disrupting clusters. The appropriate value of k was estimated to be 10 using hierarchical clustering, but a range of values of k from 8 to 13 where tested. These k values are also around the size of the likely number of major cell types in muscle and the values of k = 10 and k = 13 were chosen to represent the spread of clustering results obtained (Figure 3). GO analysis was used to identify biological processes enriched in each cluster.

In all four analyses a large cluster significantly enriched in both “cell cycle process” and “ECM organization” GO terms (*Q* < 10^−7^) and containing all of the cell cycle and part of the ECM 5 gene sets was identified (Table 2). This is consistent with the co-expression relationship of the cell cycle and ECM 5 gene sets (Figure 1A, 1B). Thus the expression of the cell cycle 5 gene set is correlated with the ECM organization process across cattle muscle development. Of the remaining clusters, one cluster was identified in which the genes were enriched in the “angiogenesis” GO term in three analyses, and in all of the three analyses were also enriched in the “ECM organization” GO term (Figure 3). Similarly, another cluster was identified in which the genes were enriched in the “angiogenesis” GO term in all four analyses, and in three of the four analyses also enriched in the “regulation of locomotion” GO term (Figure 3). A full list of the genes present in each of the groups identified by a k-means cluster and a GO term is contained in the supplementary material (Additional file 3: Table S2).

In order to determine the relationships between these GO terms, the genes appearing more than three times in the same GO terms from the equivalent clusters in all four k-means clustering analyses were investigated (Table 2). Subtle differences in expression profiles were identified for each 5 gene set (Figure 1). The ECM2 5 gene set expression profile is different from the ECM1 5 gene set profile as it is relatively constant across the prenatal samples (Figure 1C), rather than declining (Figure 1D). In contrast, the profile of the angiogenesis 5 gene set has a small increase in expression across the prenatal samples and the locomotion gene set has a large increase in expression across the prenatal samples (Figure 1E, 1F). This is consistent with the timing of the development of blood vessels in mammalian muscle [32].

In our previous Always-Correlated network based on the cattle development dataset and another independent dataset derived from a diet restriction experiment a module of genes (53 including the cell cycle 5 gene set), which included the smaller module previously annotated as “cell cycle”, was identified [15]. A large cluster of genes (Additional file 4: Table S3) enriched for the “extracellular matrix organization” GO term which contained all genes in the ECM1 5 gene set but only one gene in the ECM2 5 gene set, was adjacent to the cell cycle module [15] and was linked in the network (Additional file 5: Figure S2), again supporting a close link between the expression of the cell cycle genes and a subset of ECM genes.

In conclusion, whilst there are clearly different profiles of expression of subsets of the ECM genes prenatally, with one set (ECM1) being grouped with the cell cycle gene set, this is not so clear cut postnatally with more similarity between the expression of the sets of ECM, angiogenesis and cell cycle genes than prenatally.

### Linking gene expression and cell populations

Cell markers are useful tools for the identification of component cells in populations of cells, hence we reviewed the published potential markers of cells with mitotic potential (including pericytes, FAPs, satellite cells, endothelial cells and preadipocytes) and mature cells (including myotubes and adipocytes) in mammalian skeletal muscle (Table 3). Those markers which were included in the set of 5,596 genes were mapped in the cluster analysis output (Figure 3). The type 1 pericyte/FAPs surface marker *PDGFRA* (*CD104A*) [28,33] was located in the same cluster as the “cell cycle” GO term in three of the four analyses, suggesting that the FAPs lineage (includes type 1 pericytes and FAPs [28]) was a major contributor to the cell cycle gene expression signal. In addition, the gene encoding the fibroblast activation protein (FAP), expressed by fibroblasts associated with ECM-remodelling [34], was always located in the same cluster as *PDGFRA*.

A number of the endothelial cell markers *CDH5* (*CD144*) and *TEK* (*CD202B*) [35,36], were frequently clustered with the “angiogenesis” GO term, suggesting that in cattle LM their primary site of expression was vascular endothelial cells. Interestingly, although *CD34* has been reported to be expressed by many cell types in muscle [37], including endothelial cells (Table 3), it was the only cell marker analysed that was always associated with the “angiogenesis” GO term, suggesting that in cattle LM its most prominent site of expression is vascular endothelial cells. *VEGFC*, *NES*, *PDGFRB* (*CD140B*) and *SOX8* were also clustered with the “angiogenesis” GO term in three of the four analyses, suggesting that their most prominent sites of expression are also vascular endothelial cells. Given the likely contribution of endothelial cells to the total muscle cell population [21], it is not surprising that the endothelial cell expression of genes expressed in a number of smaller cell populations may dominate the gene expression signal. Interestingly, *ENG* (*CD105*)*,* a major glycoprotein of the vascular endothelium involved in the endothelial cell migration process [38], was located in cluster 3 with the locomotion 5 gene set, suggesting the “regulation of locomotion” GO term likely reported a cell migration activity of vascular endothelial cells. We also saw enrichment for the GO term angiogenesis in the same cluster, albeit of borderline significance. Whilst *SOX8* has been reported to be a satellite cell marker in muscle [39], on the basis of our results interpreting changes in expression of *SOX8* as changes in satellite cell numbers, as we have previously done [40], may not be appropriate.

A group of three genes, *PAX7*, *ITGB1* (*CD29*) and *CSPG4* (*NG2*) was located in the same cluster in all four analyses (Figure 3), suggesting a close relationship between these genes. The clusters containing this group of genes were not consistently enriched for GO terms. However, *ITGB1* and *CSPG4* are reported to be involved in the cell adhesion and cell signalling processes [41,42], whilst *PAX7* is a well-defined myogenic stem cell marker. The clustering of *PAX7, ITGB1* and *CSPG4*, and the lack of association of *ITGB1* and *CSPG4* with the angiogenesis related cluster, suggest that in cattle LM the major site of expression of *ITGB1* and *CSPG4* may be satellite cells, or in a process closely associated with the satellite cells.

### The Fibro/adipogenic lineage may be the major source of cell cycle gene expression

In order to explore the potential relationship between FAPs and cell cycle genes by another route, consensus gene lists were separately constructed for the cell cycle-associated “ECM organization” GO term (all genes in at least three of the four analyses) and the angiogenesis-associated “ECM organization” GO term (all genes in at least two of the three analyses) (Additional file 6: Table S4). The two lists were used to search for enrichment in any of the gene sets available on the GESA website [43]. Notably for the cell cycle-associated ECM1 genes the most significant overlap (*P < 10*^*−17*^) was with the genes more highly expressed in *CD31*^*-*^*CD34*^*+*^*CD105*^*-*^*CD45*^*-*^ cells than in *CD31*^*+*^*CD34*^*+*^*CD105*^*-*^*CD45*^*-*^ cells isolated from human adipose tissue [44]. FAPs would be expected to be in the *CD31*^*-*^ (*PECAM*^*-*^) cells (Table 3). Thus the enrichment analysis is also consistent with the FAPs and related cells being the primary source of the cell cycle genes.

### The increase in cell cycle gene expression post 12 months may be driven by adipogenesis

The expression of the cell cycle genes increases after 12 months, although ADG/kg liveweight is still decreasing [45]. Since the most likely source of the cell cycle genes is FAPs, the increase in cell cycle gene expression after 12 months is probably due to increased FAPs division activity. Activated FAPs can enter either the fibrogenic or the adipogenic lineage [28] (Figure 2). Between genotypes there was no significant difference in the expression level of the cell cycle 5 gene set, suggesting that the number of proliferating FAPs was fairly similar in the two genotypes. However, we found a large and consistent divergence in the ECM and ECM1 5 gene sets between WxH and PxH genotypes during this period (Figures 1B, 1C) and an increasingly large difference in expression of the fat deposition related genes (represented by the IMF 5 gene set [12]), all higher in the WxH than the PxH animals (Figure 1H). Both of these observations suggest that more FAPs might be expected to enter both the fibrogenic and adipogenic lineages in WxH than PxH animals. How can these apparently conflicting observations be reconciled? To address these questions we investigated the expression of potential markers of the number of preadipocytes and adipocytes (Figure 1H). The simplest explanations are that WxH and PxH animals have similar numbers of fibroblasts and adipocytes derived from FAPs, but that both cell types are more active in WxH animals at some time points, or that the same number of FAPs were generated in both genotypes, but fewer differentiated into the fibrogenic and/or adipogenic lineages in the PxH animals than in the WxH animals.

In our previous study, *WISP2* was identified as a potential marker of the preadipocyte differentiation process in cattle LM [46]. Recent research in humans and rodents has revealed that WISP2 plays a key role in regulating the commitment process from adipogenic progenitors to preadipocytes in abdominal and subcutaneous adipose tissues [47,48]. Briefly, WISP2 binds to ZNF423 to prevent PPARγ activation in the adipogenic progenitors [47]. In the cattle development dataset, *WISP2* has a unique expression profile out of nearly 20,000 genes [46] (Figure 1G). The increase in expression of *WISP2* occurs many months prior to the increase in cell cycle gene expression (Figure 1A, 1G). Thus whether the increase in *WISP2* expression is due to activation of pre-existing cells, or to the generation of a new population of cells, it appears that the FAPs derived preadipocytes may contribute little to the observed expression level of the cell cycle genes.

*ZNF423* is also more highly expressed in WxH than PxH animals from 20 to 30 months (*P* < 0.05) (Figure 1H). However, in addition to expression in preadipocytes *ZNF423* is expressed in mature adipocytes in mice [49] and the postnatal increase in expression of *ZNF423* is consistent with the appearance of adipocytes in cattle muscle [13]. *RAI14* (Retinoic Acid Induced 14) has also been proposed to be a possible marker of the early stages of adipogenesis in cattle muscle [46]. It is also more highly expressed in WxH than PxH animals at 7, 20 and 30 months (Figure 1J). It was located in the same cluster as *WISP2* in three of the four k-means clustering analyses (Figure 3). The expression profiles of *ZNF423* and *RAI14,* if they are primarily expressed by FAPs derived preadipocytes in cattle LM, also suggest that the FAPs derived preadipocytes may contribute little to the observed expression level of the cell cycle genes.

The increase in expression of the cell cycle genes post 12 months is more consistent with it being a response to increased deposition of IMF than being the driver of increased IMF deposition. This response being increased fibroblast numbers in both genotypes, with ECM deposition activity influenced by the deposition of IMF.

### Reanalysis of the impact of combined trenbolone acetate and 17β-estradiol (HGP) on key biological processes

In the light of the analysis of the PxH and WxH time course data we applied the genes and five gene sets identified above to the Brahman dataset. In the Brahman steers, expression of the cell cycle, locomotion, angiogenesis and all the ECM-related (ECM, ECM1 and ECM2) five gene set genes were significantly increased in the HGP treated groups (*P* values for most gene sets were less than 10^−8^, but for the locomotion 5 gene set was 0.002), whilst no consistent effect of HGP treatment on the expression level of either *PAX7* or *MYF5* was observed. Moreover, expression of the angiogenesis (0.87) and ECM 5 gene sets (0.79) were both correlated with expression of the cell cycle 5 gene set. However there was no correlation between expression of either *PAX7* (0.11) or *MYF5* (−0.24) and the cell cycle 5 gene set. As discussed above *SOX8* does not appear to be a reliable marker for muscle satellite cell division in cattle LM. Therefore, in contrast to the conclusions of our previous analysis [17], the increase in expression of the cell cycle genes observed in the HGP-treated cattle appears more likely to be due to an increase in the division of cells in the FAPs and/or endothelial lineages than from increased division of cells in the muscle satellite cell lineage.

HGP treatment generally reduces IMF% in cattle [50] and expression of the IMF 5 gene set was significantly reduced in the HGP treated Brahmans [12]. In contrast, HGP treatment induced a highly significant increase in *WISP2* expression (*P <* 10^−8^) [40], but reduced IMF deposition [17], the opposite of what would be predicted based on the analysis of expression of *WISP2* and the IMF 5 gene set genes in WxH and PxH genotype animals. *WISP2* expression has also been reported to be increased in a number of tissues in response to treatment with estrogens [51], testosterone [52], and other promoters of cell proliferation. Does the change in *WISP2* expression due to HGP treatment play a role in the change in deposition of IMF? One mechanism could be through increased expression of *WISP2* per cell leading to increased inhibition of the differentiation pathway from FAPs to adipocytes, fewer adipocytes and hence less deposition of IMF. However, HGP treatment had no significant effect on the expression of *ZNF423*, suggesting that there was no effect on adipocyte number, or that the effect was very small. The observations are more consistent with a direct inhibition of lipid deposition in existing adipocytes. Such an activity of secreted *WISP2* has been reported [48] and is also consistent with the increase in *WISP2* expression observed as fat deposition rates decline in the older WxH and PxH genotype animals (Figure 1E). However, direct inhibition of IMF synthesis in HGP-treated animals could also be explained by estrogen signalling, ERalpha agonists have been shown to inhibit *de novo* lipogenesis *in vitro* [53] and estradiol treatment has been shown to decrease lipogenesis and TAG storage *in vivo* [54]. Clearly further investigation of the mechanism of the suppression of lipid deposition in cattle LM by HGP is required.

## Conclusions

In postnatal LM in cattle the major source of expression of the cell cycle genes appears to be the FAP lineage, and primarily the fibroblast component of the lineage. The other cell types in the muscle, although also dividing, appear to contribute a small proportion of the signal. It is unlikely that the fibroblasts are the drivers of muscle growth, rather that they are responding to the remodelling requirements of hypertrophic growth of the contractile cells and the deposition of lipid in the intramuscular adipocytes. The similar expression profiles of the ECM and angiogenesis genes is consistent with the formation of a scaffold supporting the growth of the contractile and adipose cells (Figure 2).

We have proposed gene sets and gene markers for many of the major cell types and biological processes in cattle LM which can be used to describe the status of the main components of cattle skeletal muscle under various conditions. Whilst further validation is required these tools should enable the improvement of muscle ADG and the design of more efficient feeding strategies in the beef cattle industry. By monitoring expression levels of these gene sets, the impact of different diets on muscle composition and growth rate can be estimated from a single sample. Utilisation of the cell cycle 5 gene set to estimate ADG/kg muscle mass from a single observation will require knowledge of other aspects of the age and growth of the animals. The approach and the gene sets described are likely to be applicable more generally to the study of mammalian muscle growth.

## Methods

Use of the animals and the procedures performed in this study were approved by the Industry & Investment New South Wales (NSW) Orange Agriculture Institute Animal Ethics Committee, Commonwealth Scientific and Industrial Organisation (CSIRO) Rockhampton Animal Experimentation Ethics Committee, and the Department of Agriculture and Food, Western Australia (WA) Animal Ethics Committee.

### Gene expression datasets

The design of the experiment and the generation of the gene expression data from the 48 Brahmans have been described in detail previously [17,55]. In brief, 48 Brahman steers were fed concentrated feed at two different sites, Western Australia (WA) and New South Wales (NSW), with or without HGP-treatment (200 mg trenbolone acetate, 20 mg 17β-estradiol) at each site. These animals were slaughtered at an average age of 20 months (range 17 to 23 months). Gene expression data was generated from mRNA purified from LM biopsy samples on the Agilent Bovine microarray platform. As previously reported [56], a combination of ANOVA models and mixtures of distributions were employed to normalize expression signals. In detail, gene expression data normalization was achieved by fitting the following ANOVA mixed-effect model: Y_*ijftmn*_ = μ + A_*i*_ *+* G_*m*_ + AG_*im*_ + LG_*fm*_ + MG_*jm*_ + TG_*tm*_ + e_*ijtmn*_, where Y_*ijkftmn*_ represents the n-th background-adjusted, base-2 log-intensity reading from the *i*-th array and corresponding to the *m*-th gene (or Agilent probe) at the *t*-th hormone treatment (HGP or -HGP) taken from an animal raised in the *f*-th location (NSW or WA) with the *j*-th marker genotype (1, 2, or 3); μ is the overall mean; G represents the random gene effects; AG, LG, MG, and TG are the random interaction effects of array gene, location gene, marker genotype gene, and hormone treatment gene, respectively; and e is the random error term. Using standard stochastic assumptions, we assumed the effects of G, AG, LG, MG, TG, and e to be independent realizations from a normal distribution with zero mean and between-gene, between-gene within-array, between-gene within-location, between-gene within maker genotype, between-gene within-hormone treatment, and within-gene components of variance, respectively. Restricted maximum likelihood estimates of variance components and solutions to model effects were obtained using the analytical gradients option of VCE6 software [57]. The solutions to the TG effect were used as the normalized mean expression of each gene in each of the conditions under scrutiny. The data is available from NCBI GEO with the accession number GSE25005.

The design of the experiment and the generation of the gene expression data from the Wagyu x Hereford (WxH) and Piedmontese x Hereford (PxH) genotype animals have been described in detail previously [20,58]. Briefly biopsy samples of LM were collected from nine development stages (pre and postnatal) and a post slaughter sample. Sampling ages were: 60, 135 and 195 days post conception, at birth (~280 days) and 3, 7, 12, 20, 25 and 30 months of age. From 60 days to birth the samples were from different animals, thereafter the samples were serial biopsies from the same individuals. The animals were weaned at 7 months and were fed on grass to 25 months and concentrate to 30 months [55]. For each stage, gene expression values were collected for three or four individuals on the Agilent Bovine microarray platform, as reported previously [59]. The data was normalized using a linear ANOVA mixed-model as previously described [60]. Briefly, The Multivariate mixed-model equations of ANOVA models were employed to make full use of the information available, with multiple factors and a hierarchy of sources of variation. The proposed model is equivalent across experiments, although different gene variances across experiments, and across components within experiment, are allowed. In matrix notation, and for the *i*th experiment (*i* = 1, 2, 3) and the *j*th design component within experiment (*j* = 1, 2 for *i* = 1 and 3; and *j* = 1, 2, 3 for *i* = 2) the following model was fitted: **y***ij* = **X***ij***β***ij* + **G***ij***g***ij* + **A***ij***a***ij* + **V***ij***v***ij* + **e***ij* where **y***ij* is the (n*ij* × 1) vector of signals from the *i*th experiment and the jth design component within experiment; n*i* is the size of **y***ij*; **X***ij* is an (n*ij* × p*ij*) incidence matrix relating the p*ij* fixed comparison group effects in **β***ij* with signals in **y***ij*; **G***ij* is an (n*ij* × g) matrix relating the random gene (or array elements) effects in **g***ij* with **y***ij*; **A***ij* is an (n*ij* × a*ij*) matrix relating the random gene × array-printing-block interaction effects in a*ij* with **y***ij*; **V***ij* is an (n*ij* × v*ij*) matrix relating the random gene × variety effects in **v***ij* with **y***ij*; and **e***ij* is the (n*ij* × 1) vector of random errors. The data is available as supplementary material for [14] and from NCBI GEO with the accession number GSE44030.

### Statistics and bioinformatics

The ADG/kg liveweight was calculated as follows: ADG (from HGP implant, or equivalent time point, to slaughter) of the Brahman steers was divided by the average body weight at the two time-points: HGP implant (or equivalent time point) and at slaughter. The correlation between gene expression and the phenotypic measurements of the Brahman cattle was calculated using the “CORREL” function in Microsoft Excel.

Student’s t-test of significance was calculated using the “TTEST” function (two tailed and assuming unequal variance of the two distributions) in Microsoft Excel. The normality of the distribution of values in both the Brahman dataset and development dataset was checked using “1-sample K-S test” function in nonparametric test category of SPSS 19.0. The t-test was applied to compare the differences in the expression of single genes and the five gene sets between groups of Brahman steers, or between PxH and WxH cattle at the same or different time points.

Gene ontology term enrichment analysis was carried out by using GOrilla [16]. This network tool uses a hypergeometric statistic to quantify functional enrichment in ranked gene lists [61]. *P*-values, and the false discovery rate (FDR) *Q*-values calculated using the Benjamini and Hochberg method [62], were provided in the results output of the GOrilla website.

Z-scores were used to reduce the impact of differences in levels of expression and dynamic range of expression of genes on combining the gene expression data from two or more genes. The gene expression data (log2) for each gene was rescaled to a mean of 0 across the whole set of the Brahmans, or throughout development and including both the WxH and PxH genotype animals for the developmental time course. The difference from the mean of each measurement (a single gene in a single animal) was divided by the relevant standard deviation for the gene using Microsoft Excel. The standard deviation of the five gene sets in a subset of animals was calculated based on the average z-score of the gene expression values of the five genes in a gene set by using the corresponding formula in Microsoft Excel.

Cluster analysis was carried out by using the “K-Means Cluster” function in SPSS 19.0 with default settings and two k-values: 10 and 13. The appropriate value of k was estimated using hierarchical clustering. All the cluster analyses were based on the z-score of the gene expression values in the LM from the PxH and WxH genotype animals across development (all ten time points) or the seven postnatal time points and across the two genotypes at the same time.

To evaluate the significance of the relationship between the identified five gene sets and the phenotype for the Brahman animals the correlation of a defined five gene set was compared with the correlation of random gene sets. Random sampling was carried out in MATLAB R2012a using custom scripts. Sets of five randomly selected genes were sampled 100,000 times from the rescaled Brahman dataset. The number of gene combinations possessing higher correlation with ADG/kg liveweight in the Brahman dataset than the defined five gene sets were counted.

## Availability of supporting data

Supporting data is available in the supplementary files and the gene expression data is available from GenBank, GSE25005 [63] and GSE44030 [64].

## Additional files

Additional file 1: Table S1. List of correlation between gene expression and ADG/kg liveweight in Brahman. Additional file 2: Figure S1. The expression profiles of *PAX7* and *MYF5* through development in PxH and WxH cattle. The gene expression values are replaced by the corresponding z-scores. Additional file 3: Table S2. Detailed information of k-means cluster analysis. k = 13 with all timepoints, k = 13 with postnatal timepoints, k = 10 with all timepoints and k = 10 with postnatal timepoints are shown in sheet1, sheet 2, sheet 3 and sheet 4 respectively. Additional file 4: Table S3. Gene list of the cell cycle module and related genes from the Always-Correlated network. Additional file 5: Figure S2. The “cell cycle” module and genes closely related to this module in the Always-Correlated network from our previous study [15]. The whole group of 246 genes (Additional file 3: Table S2) were significantly enriched in two GO terms: “ECM organization” (Q < 10^−11^) and “cell cycle process” (Q < 10^-8^), which were indicated in blue and red respectively. Additional file 6: Table S4. List of overlap of ECM-related genes based on the cluster analysis. The consensus gene lists for the cell cycle-associated “ECM organization” GO term (all genes in at least three of the four analyses) and the angiogenesis-associated “ECM organization” GO term (all genes in at least two of the three analyses) are shown in sheet 1 and sheet 2 separately.

## Abbreviations

ADG: Average daily gain
ECM: Extracellular matrix
EMA: Eye muscle area
FAPs: fibro/adipogenic progenitors
GO: Gene ontology
IMF: Intramuscular fat
LM: Longissimus muscle
NSW: New South Wales
PxH: Piedmontese x Hereford
TAG: Triglyceride
WxH: Wagyu x Hereford
WA: Western Australia
