# Supplementary figures and images for: Transcriptome analysis of cattle muscle identifies potential markers for skeletal muscle growth rate and major cell types
Source: BMC Genomics. 2015 Mar 13;16(1):177. doi: 10.1186/s12864-015-1403-x (PMC4364331; doi:10.1186/s12864-015-1403-x)

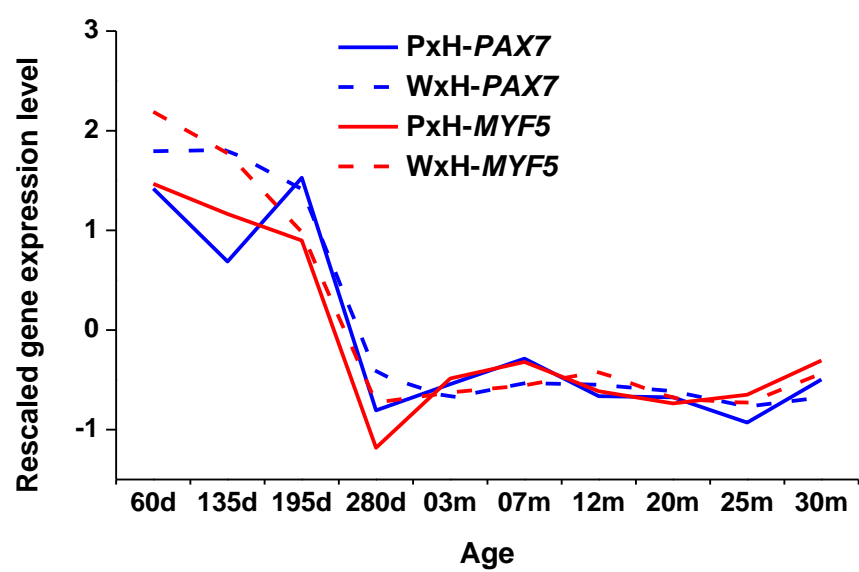

Supplement: Additional file 2: Figure S1. — The expression profiles of PAX7 and MYF5 through development in PxH and WxH cattle. The gene expression values are replaced by the corresponding z-scores. [file 12864_2015_1403_MOESM2_ESM.pdf]

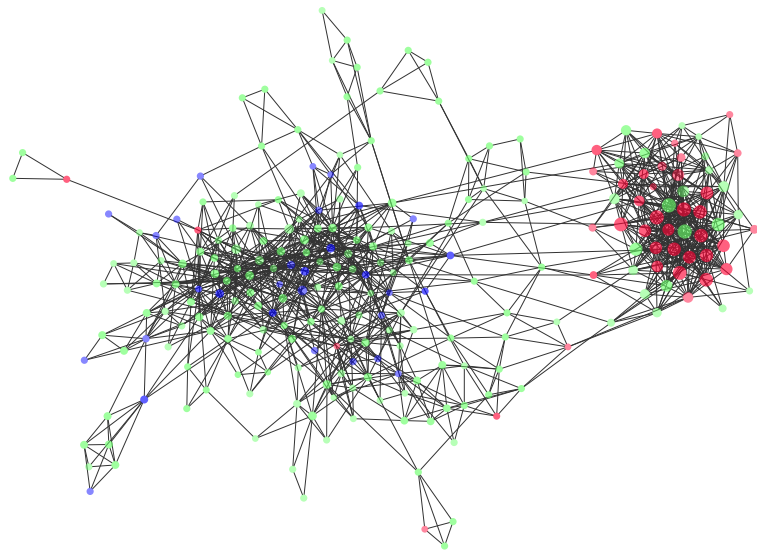

Supplement: Additional file 5: Figure S2. — The “cell cycle” module and genes closely related to this module in the Always-Correlated network from our previous study [15]. The whole group of 246 genes (Additional file 3: Table S2) were significantly enriched in two GO terms: “ECM organization” (Q < 10−11) and “cell cycle process” (Q < 10-8), which were indicated in blue and red respectively. [file 12864_2015_1403_MOESM5_ESM.pdf]
